# Correction: Sensitive immunosensing of α-synuclein protein in human plasma samples using gold nanoparticles conjugated with graphene: an innovative immuno-platform towards early stage identification of Parkinson’s disease using point of care (POC) analysis

**DOI:** 10.1039/d2ra90011d

**Published:** 2022-02-16

**Authors:** Esmaeil Darvish Aminabad, Ahmad Mobed, Mohammad Hasanzadeh, Mohammad Ali Hosseinpour Feizi, Reza Safaralizadeh, Farzad Seidi

**Affiliations:** Department of Biology, Faculty of Natural Sciences, University of Tabriz Tabriz Iran MH-Faizi@ea-sciencepark.org.ir; Pharmaceutical Analysis Recent Center, Tabriz University of Medical Sciences Tabriz 51664 Iran Hasanzadehm@tbzmed.ac.ir; Physical Medicine and Rehabilitation Research Center, Tabriz University of Medical Sciences Tabriz Iran; Jiangsu Co-Innovation Center for Efficient Processing and Utilization of Forest Resources and International Innovation Center for Forest Chemicals and Materials, Nanjing Forestry University Nanjing 210037 China

## Abstract

Correction for ‘Sensitive immunosensing of α-synuclein protein in human plasma samples using gold nanoparticles conjugated with graphene: an innovative immuno-platform towards early stage identification of Parkinson’s disease using point of care (POC) analysis’ by Esmaeil Darvish Aminabad *et al.*, *RSC Adv.*, 2022, **12**, 4346–4357, DOI: 10.1039/D1RA06437A.

Affiliation a was incorrectly given in the original article. The correct affiliation is as shown in this Correction notice.

The Royal Society of Chemistry apologises for these errors and any consequent inconvenience to authors and readers.

## Supplementary Material

